# Association of dopaminergic pathway gene polymorphisms with chronic renal insufficiency among Asian Indians with type-2 diabetes

**DOI:** 10.1186/1471-2156-9-26

**Published:** 2008-03-22

**Authors:** Pushplata Prasad, KM Prasanna Kumar, AC Ammini, Arvind Gupta, Rajeev Gupta, BK Thelma

**Affiliations:** 1 Department of Genetics, University of Delhi South Campus, New Delhi, India; 2 Department of Endocrinology and Metabolism, M.S. Ramiah Medical College, Bangalore, India; 3 Department of Endocrinology, All India Institute of Medical Sciences, New Delhi, India; 4 Jaipur Diabetes and Research Centre, Jaipur, India; 5 Monilek Hospital and Research Centre, Jaipur, India

## Abstract

**Background:**

Genetic markers conferring susceptibility to diabetes specific renal disease remains to be identified for early prediction and development of effective drugs and therapies. Inconsistent results obtained from analysis of genes from classical pathways generate need for examination of unconventional genetic markers having role in regulation of renal function. Experimental and clinical evidences suggest that dopamine is an important natriuretic hormone. Therefore, various genes involved in regulation of dopamine bioavailability could play a role in diabetic chronic renal insufficiency (CRI). We investigated the contribution of 12 polymorphisms from five Dopaminergic pathway genes to CRI among type-2 diabetic Asian Indian subjects.

**Methods:**

Genetic association of 12 polymorphisms (SNPs) from five genes namely-dopamine receptor-1 (*DRD1*), *DRD2*, *DRD3*, *DRD4*, andcatechol-O-methyltransferase (*COMT*) with diabetic CRI was investigated using a case-control approach. Logistic regression analysis was carried out to correlate various clinical parameters with genotypes, and to study pair wise interactions between SNPs of different genes.

**Results:**

SNPs *-141 ins/del C and G>A *(1 kb upstream from exon 2) in DRD2 gene showed significant allelic and genotypic association. Allele *-141 insC *and genotype *-141 insC/insC *of *-141 ins/del C *polymorphism, and allele A of *G>A *SNP were found to be predisposing to CRI. Our result of allelic and genotypic association of -*141 insC/delC *SNP was also reflected in the haplotypic association. Heterozygous genotype of polymorphism *900 ins/del C in COMT *gene was predisposing towards CRI.

**Conclusion:**

Some polymorphisms in *DRD2 *and *COMT *genes are significantly associated with susceptibility to CRI in the Asian Indian population which, if confirmed would be consistent with a suggested role of dopamine metabolism in disease occurrence.

## Background

A large number of genetic association studies for unraveling the genetic basis of renal disease in diabetes have been carried out using polymorphisms in candidate genes from various postulated biochemical and metabolic pathways. These include renin-angiotensin-aldosterone system (RAAS), protein kinase C (PKC)-polyol, advanced glycation end (AGE) product, hexosamine and oxidative stress. However the results are confusing and identification of genetic susceptibility to chronic renal insufficiency (CRI) continues to be a challenge. This suggests the need for examining other unconventional genetic markers having role in regulation of normal physiological functions of kidney, especially under hyperglycemic condition.

Dopamine is an important natriuretic hormone involved in the regulation of blood pressure, and salt-water reabsorption in kidney. While angiotensin II, another hormone involved in natriuresis, stimulates sodium reabsorption through activation of Na-K-ATPase and Na/H exchanger, dopamine acts antagonistically and inhibits reabsorption by inhibiting Na-K-ATPase and Na/H exchanger, regulating sodium transport and excretion in the proximal tubule [1-3]. Moreover, there are ample empirical evidences to support that dopamine through specific dopamine receptors influences various components of renin angiotensin aldosterone system (RAAS). While D1 like receptors stimulate renin release, D3 receptor inhibits it [4]. D3 receptor of dopamine decreases angiotensin II type 1 receptor (*AT1*) expression and angiotensin II binding to *AT1 *in renal proximal tubules [3,4]. Disruption of D3 receptor gene in mice induces a renin dependent form of hypertension [5]. Such interactions between dopamine and components of RAAS could modulate the potential effects of RAAS, which besides regulating vascular tone and Na ion balance in kidney also influences cytokine mediated inflammatory pathway in diabetic kidney disease [6-9]. In addition, administration of nitecapone, an inhibitor of dopamine metabolizing enzyme catechol-O-methyltransferase (*COMT*; EC 2.1.1.6), has been found to effectively abolish glomerular hypertension and reduce progression to glomerulosclerosis by inhibiting Na-K-ATPase in a dopamine dependent fashion [10,11]. These findings reiterate a potential role of dopaminergic pathway genes in conferring susceptibility to diabetic CRI through modulation of dopamine levels.

Several polymorphisms in dopaminergic pathway genes have been identified and extensively investigated for their role in hypertension and schizophrenia [12-17]. There is only one report on association of D3 receptor of dopamine with diabetic nephropathy but in individuals with type-1 diabetes [18]. Due to significant role of genes from dopaminergic pathway in kidney function, in this pilot study, we investigated the susceptibility conferred by polymorphisms in dopaminergic pathway genes to CRI among individuals with type-2 diabetes, hitherto unexplored.

## Methods

### Subjects

Complete clinical and demographic characteristics of the study population have been previously reported [19]. Briefly, in this retrospective case-control analysis consecutive subjects suffering from type-2 diabetes with CRI (cases, CRI, n = 196), and diabetics without any evidence of diabetic kidney disease (controls, DM, n = 225) were recruited from the outpatient departments of the four participating medical institutions situated across India. The study was approved by respective institutional ethics committee and an informed consent obtained from all the participants. For CRI, the inclusion criteria were subjects with type 2 diabetes for ≥2 years, plus two or more of the following: serum creatinine ≥150 umol/l, urinary albumin excretion rate (UAER) > 200 mg/l, and presence of retinopathy. Retinopathy was diagnosed by either fundoscopic examination or fluoroangiographic study. 10 ml venous blood was collected from each individual included in the study for biochemical and genetic analysis. Biochemical analyses to determine fasting glucose, glycated hemoglobin, serum creatinine, triglycerides, total cholesterol, and albumin were carried out at the respective centres using automated analyzers. Using serum creatinine as a surrogate marker we calculated glomerular filtration rate (GFR) by using the online modified diet in renal disease (MDRD) calculator. Patients with drug induced nephrotoxic damage or secondary causes of albuminuria such as obstructive renal disease, renal stone disease and acute urinary tract infection were excluded from this group. Normoalbuminuric (AER < 20 mg/l) type-2 diabetes subjects of ≥ 10 years duration of diabetes (mean duration 17.07 ± 6.69 years) were recruited as control diabetes subjects. Normoalbuminuria among control (DM) subjects was determined by timed urine collections. An aliquot of blood from the four centres was transported to the genetics laboratory for DNA isolation. DNA was isolated from the lymphocytes using the conventional phenol-chloroform organic extraction method and used for genetic analysis.

### Genetic analysis

A total of 12 polymorphisms from five candidate genes were genotyped in DM (controls) and CRI (cases) subjects. These include:

***COMT *(+116790; 22q11.21)**: *-287 A>G *in promoter [14]; *408 C>G*) [20]*and 472 G>A (Val 158 Met) *[14] in exon 4 and *900 ins/del C *in 3'UTR [15];

*DRD1 *(*126449; 5q35.2): *-48 A>G *(5'UTR of exon 2) (**rs4532**) [2];

*DRD2 *(*126450; 11q23.2): *-141 ins/del C *(**rs1799732**)*; G>A, 1 kb upstream from exon 2 *(rs17294542) [21,22]; and *T>C, 10 kb downstream from exon 8 *(rs1800497) [21,22];

*DRD3 *(*126451; 3q13.31): -rs6280 *C>T (ser9Gly) *in exon 2 [16];

*DRD4 *(*126452; 11p15.5): -*120 bp duplication*, 1.2 kb upstream from initiation codon [23], *-521 C>T *[17]; and 48 bp VNTR in exon 3 [24].

Genotyping was done using either polymerase chain reaction (PCR)-restriction fragment length polymorphisms (RFLP) or PCR-length polymorphism (LP) approach following the method described in the respective quoted references. Genotypic profiles obtained for each of the polymorphisms are presented in Additional file 1. The digested PCR products were resolved on 2–3% agarose gel stained with ethidium bromide.

### Statistical Analysis

Comparison of all clinical variables between DM and CRI subjects were carried out by χ^2 ^test for nominal variables or t-test for continuous variables. Continuous variables where skewed distribution was observed were compared by Mann Whitney U test and values are reported as mean and range. Hardy-Weinberg equilibrium (HWE) was tested for each of the genetic marker. Allelic and genotypic associations of SNPs/VNTRs were evaluated by Pearson's χ^2 ^test followed by odds ratio and 95% CI computation. Power of the sample size for each of the SNPs was calculated using PAWE software version 1.2 [25,26]. Haplotype analysis was performed using PHASE-standard analysis version 2.0.2 [27,28]. Chi-square values were derived from a series of 2 × 2 contingency tables based on the frequency of each haplotype versus all others between the DM and the CRI groups. Linear and logistic regression analyses were carried out to correlate various clinical parameters with genotypes and to study pair wise interactions between SNPs of different genes. P values < 0.05 were subject to Bonferroni's correction.

## Results

### Clinical analysis

Clinical characteristics of the two patient groups are presented in Table 1.

**Table 1:** Clinical characteristics of the study population

**Characteristics**	**DM^a ^(n = 225)**	**CRI^b ^(n = 196)**	**P**
Gender (M/F)	76/149	65/131	0.89^d^
Age (years)	60.6 ± 11.5	57 ± 12.8	0.10^c^
BMI (kg/m2)	23.1 ± 0.3	23.4 ± 0.2	0.00
**Duration of Diabetes (years)**	**17.07 ± 6.69**	**10.4 ± 7.7**	**<0.05^c^**
Hb A_1c _(%)	7.3 ± 1.0	7.5 ± 1.1	0.949^c^
Systolic pressure (mm Hg) *	140 (106–190)	150 (110–210)	0.002^e^
Diastolic pressure (mm Hg) *	84 (80–104)	90 (70–110)	0.025^e^
Serum creatinine (μmol/l) *	**84 (35–108)**	**177 (124–1112)**	**<<0.05^e^**
UAER (mg/liter)*	**10 (1–16)**	**864 (320–1584)**	**<<0.05^e^**
**GFR (mls/min/1.73 m^2^)**	**81.44 ± 21.85**	**27.83 ± 24**	**<<0.05^c^**
Serum triglyceride (mmol/l)	1.7 ± 0.67	1.8 ± 1.4	0.307^c^
Serum cholesterol (mmol/l)	4.8 ± 0.97	4.9 ± 0.92	0.852^c^
**Retinopathy (%) **Non-proliferative (%) Proliferative (%)	**24 **15 09	**88 **35 53	**<<0.05^d^**
Cardiovascular events (%)	3	8.3	

Data presented as mean ± SD (* median and range). ^a^type-2 diabetes subjects without nephropathy (DM); ^b ^with diabetic renal insufficiency (CRI); ^c ^Student's t test; ^d ^Pearson's χ^2 ^test; ^e ^Mann-Whitney U test.

### Genetic analysis: Efficiency of PCR based genotyping was approximately 98% with at least two additional attempts for genotyping of failed samples

#### COMT

No allelic association of any of the four SNPs in this gene with diabetic CRI was observed (Table 2). Only the heterozygote genotype of *900 ins/del C *SNP was significantly associated with CRI (P = 0.018, OR 1.66, 95%CI 1.09–2.52). Haplotypes generated using genotypic profiles of the four SNPs in COMT gene are presented in Table 3. We observed significant association of three haplotypes in COMT gene, which were retained even after Bonferroni's correction.

**Table 2:** Allele and genotype frequencies of SNPs in COMT, DRD1, DRD2, DRD3 and DRD4 genes and their association status with diabetic CRI.

**SNPs**	**Allele frequency**		**Genotype frequency**		**Association**	
	**DM (n = 225)**	**CRI (n = 196)**	**DM (n = 225)**	**CRI (n = 196)**	**Allele (df = 1)**	**Genotype (df = 2)**
COMT -287 G>A	G = 0.63	G = 0.65	GG = 0.38	GG = 0.43	χ^2 ^= 0.45;	χ^2 ^= 0.93;
	A = 0.37	A = 0.35	GA = 0.49	GA = 0.44	P = 0.50	P = 0.63
			AA = 0.13	AA = 0.13		
COMT 408 C>G	C = 0.71	C = 0.69	CC = 0.51	CC = 0.47	χ^2 ^= 0.61;	χ^2 ^= 0.98;
	G = 0.29	G = 0.31	CG = 0.39	CG = 0.44	P = 0.43	P = 0.61
			GG = 0.10	GG = 0.09		
COMT 472 G > A (Val158 Met)	G = 0.53	G = 0.59	GG = 0.28	GG = 0.38	χ^2 ^= 2.71;	χ^2 ^= 4.10;
	A = 0.47	A = 0.41	GA = 0.51	GA = 0.42	P = 0.10	P = 0.13
			AA = 0.21	AA = 0.20		
COMT 900 Ins/Del C	Ins = 0.71	Ins = 0.69	Ins/Ins = 0.54	Ins/Ins = 0.46	χ^2 ^= 0.59;	χ^2 ^= 5.83;
	Del = 0.29	Del = 0.31	Ins/Del = 0.34	Ins/Del = 0.46	P = 0.44	P = 0.054
			Del/Del = 0.12	Del/Del = 0.08		
DRD1 -48A>G	A = 0.55	A = 0.82	AA = 0.33	AA = 0.34	χ^2 ^= 0.33;	χ^2 ^= 1.83;
	G = 0.45	G = 0.18	AG = 0.44	AG = 0.46	P = 0.57	P = 0.401
			GG = 0.23	GG = 0.20		
DRD2 -141 ins/del C	Ins = 0.74	Ins = 0.85	Ins/Ins = 0.57	Ins/Ins = 0.74	**χ^2 ^= 12.11;**	**χ^2 ^= 11.3;**
	Del = 0.26	Del = 0.15	Ins/Del = 0.35	Ins/Del = 0.23	**P < 0.001^a^**	**P = 0.004^a^**
			Del/Del = 0.08	Del/Del = 0.03		
DRD2 G>A	G = 0.77	G = 0.66	GG = 0.61	GG = 0.45	**χ^2 ^= 7.94;**	**χ^2 ^= 7.83;**
	A = 0.23	A = 0.34	GA = 0.31	GA = 0.42	**P = 0.005**	**P = 0.02**
			AA = 0.08	AA = 0.13		
DRD2 T>C	T = 0.74	T = 0.69	TT = 0.55	TT = 0.48	χ^2 ^= 2.30;	χ^2 ^= 2.18;
	C = 0.26	C = 0.31	TC = 0.37	TC = 0.41	P = 0.13	P = 0.335
			CC = 0.08	CC = 0.11		
DRD3 Ser9Gly	C = 0.64	C = 0.59	CC = 0.43	CC = 0.35	χ^2 ^= 2.03;	χ^2 ^= 2.8
	G = 0.36	G = 0.41	CG = 0.42	CG = 0.48	P = 0.15	P = 0.25
			GG = 0.15	GG = 0.17		
DRD4 -120 bp Deletion	Ins = 0.68	Ins = 0.73	Ins/Ins = 0.48	Ins/Ins = 0.53	χ^2 ^= 1.77;	χ^2 ^= 1.72;
	Del = 0.32	Del = 0.27	Ins/Del = 0.42	Ins/Del = 0.39	P = 0.18	P = 0.42
			Del/Del = 0.105	Del/Del = 0.08		
DRD4 -521 C>T	C = 0.40	C = 0.45	CC = 0.18	CC = 0.22	χ^2 ^= 1.25;	χ^2 ^= 1.15;
	T = 0.60	T = 0.55	CT = 0.44	CT = 0.45	P = 0.26	P = 0.56
			TT = 0.38	TT = 0.33		
DRD4 48 bp VNTR	371 = 0.06	371 = 0.08	371/371 = 0.005	371/371 = 0.02	χ^2 ^= 7.25;	χ^2 ^= 10.6;
	419 = 0.02	419 = 0.02	467/371 = 0.10	467/371 = 0.10	P = 0.30 (df = 6)	P = 0.156 (df = 7)
	467 = 0.86	467 = 0.80	467/419 = 0.02	467/419 = 0.04		
	563 = 0.03	563 = 0.04	467/467 = 0.78	467/467 = 0.68		
	611 = 0.01	611 = 0.01	467/563 = 0.02	467/563 = 0.03		
	647 = 0.02	647 = 0.04	467/611 = 0.02	467/611 = 0.03		
			467/647 = 0.02	467/647 = 0.067		
			563/563 = 0.014	419/647 = 0.007		

^a^Significant after Bonferroni's correction α = 0.0042

**Table 3:** SNP haplotypes in COMT gene

**^a^Haplotype**	DM (393) n*;f**	CRI (372) n*;f**	**χ^2^**	**P**	**OR (95% CI)**
1111	50 (0.127)	64 (0.172)	3.18	0.074	1.44 (0.96–2.15)
**1112**	**33 (0.084)**	**04 (0.01)**	**20.48**	**^b^< 0.001**	**0.12 (0.04–0.34)**
1121	59 (0.150)	50 (0.134)	0.33	0.56	0.89 (0.59–1.33)
**1122**	**03 (0.007)**	**28 (0.075)**	**22.69**	**^b ^< 0.001**	**10.66 (3.21–35.38)**
1211	39 (0.01)	43 (0.115)	0.08	0.78	1.07 (0.67–1.68)
1212	55 (0.14)	51 (0.137)	0.005	0.94	0.98 (0.65–1.48)
1221	06 (0.015)	02 (0.005)			
2111	23 (0.058)	24 (0.064)	0.138	0.71	1.12 (0.62–2.02)
2112	00 (0.00)	04 (0.01)			
**2121**	**103 (0.262)**	**62 (0.166)**	**9.927**	**^b^0.0016**	**0.57 (0.40–0.81)**
2122	13 (0.033)	20 (0.05)	2.04	0.15	1.67 (0.82–3.41)
2211	01 (0.002)	08 (0.021)			
2212	06 (0.015)	05 (0.013)	0.04	0.84	0.89 (0.27–2.92)
2221	02 (0.005)	07 (0.019)			

^a^Order of SNPs in the COMT haplotypes: -287 A>G, 408 C>G, 472 G > A and 900 Ins/Del C

^b^Significant after Bonferroni's correction α = 0.0035

*n = total number

**f = frequency

#### DRD1

No allelic or genotypic association of the -48 A>G polymorphism with CRI in our sample set was seen (Table 2).

#### DRD2

Of the three polymorphisms analysed in this gene, we observed a highly significant allelic and genotypic association of -*141 ins/del C and G>A *(1 kb upstream from exon 2) SNPs. *T>C *(10 kb downstream from exon 8) SNP did not show any allelic or genotypic association with diabetic kidney disease (Table 2). Out of the eight possible haplotypes in our sample set, three (-141 insC-A-T, -141 insC-A-C, and -141 delC-A-C) were significantly associated with CRI (Table 4).

**Table 4:** SNP haplotypes in DRD2 gene

**^a^Haplotype**	DM (398) n*;f**	CRI (380) n*;f**	**χ^2^**	**P**	**O.R (95% CI)**
111	217 (0.545)	173 (0.046)	4.97	0.03	0.69 (0.49–0.96)
211	56 (0.14)	40 (0.105)	1.65	0.20	0.73 (0.44–1.19)
112	18 (0.045)	39 (0.102)	6.94	0.01	2.37 (1.23–4.60)
212	14 (0.035)	00 (0.00)	6.23	0.01	0.09 (0.01–0.74)
**121**	**11 (0.027)**	**38 (0.1)**	**^b^13.51**	**<<0.01**	**4.08 (1.83–9.11)**
221	01 (0.002)	01 (0.002)	0.43	0.51	1.08 (0.08–17.32)
**122**	**41 (0.103)**	**80 (0.21)**	**^b^12.94**	**<<0.01**	**2.33 (1.46–3.73)**
**222**	**39 (0.098)**	**09 (0.023)**	**^b^13.50**	**<<0.01**	**0.23 (0.10–0.54)**

^a^Order of SNPs in the DRD2 haplotypes: -141 ins/del C – G>A (Intron 1) – T>C (Intron 7)

^b^Significant after Bonferroni's correction α = 0.0063

*n = total number

**f = frequency

#### DRD3

No allelic or genotypic association of the most commonly investigated *Ser9Gly *SNP with CRI was observed in this study (Table 2).

#### DRD4

None of the three polymorphisms tested in the gene showed significant genotypic or allelic association with CRI. Out of 21 possible haplotypes in our case-control population, only haplotype 2-1-3 [(-120) no duplication – (-521) C – VNTR 467] was found to be marginally associated with CRI. However, after Bonferroni's correction the haplotype did not retain significance (Additional file 2).

Multivariate logistic regression analysis was carried out using disease status as a dependent variable, and age, gender, BMI, duration of diabetes, creatinine clearance rate and genotypes of all 12 polymorphisms as independent variables. A significant association of SNPs -141 ins/del C (P = 0.000; OR 2.85, 95%CI 1.73–4.74) and *G>A *(1 kb upstream from exon 2) (P = 0.006; OR 1.84, 95%CI 1.19–2.84) in DRD2 gene with CRI was observed. Further categorical analysis identified genotypes -141 ins/ins C (P = 0.015; OR 3.35, 95%CI 1.27–8.84) and AA (P = 0.013; OR 1.88, 95%CI 1.14–3.10) of SNPs *-141 ins/del C, and G>A *(1 kb upstream from exon 2) respectively of *DRD2 *gene to be predisposing to CRI. Pair-wise interactions between different polymorphisms included in this study were tested using multiple logistic regression analysis. No significant interaction between any of these polymorphisms/genes was observed.

## Discussion

In addition to its classical role as a neurotransmitter, dopamine also acts as a natriuretic hormone that has implications in kidney function. Dopamine down regulation causes Na retention and renal tissue injury [11]. In addition dopamine also modulates the activity and availability of Ang II, the key molecule of renin-angiotensin-aldosterone system in kidney. An altered interplay of dopamine and Ang II could disturb the vascular tone, sodium ion balance, and enhance cytokine-mediated injury to renal tissue. Thus, as mentioned earlier, genetic variations in genes controlling the bio-availability of dopamine could be pathological. While various studies have implicated genetic variations in COMT and dopamine receptor genes in essential hypertension [29-35], and one study has implicated COMT (based on a functional assay using a COMT inhibitor) in diabetic kidney disease in rats [10], an association of dopaminergic pathway gene polymorphisms with kidney disease among individuals with type-2 diabetes has not been tested in any population so far. This is the first report on the role of multiple SNPs in dopaminergic pathway genes conferring susceptibility to diabetic CRI.

Out of four SNPs tested in *COMT *gene only *900 ins/del C *showed a strong trend towards genotypic association (Table 2), with *insC/delC *genotype being predisposing (OR 1.66, CI 1.09–2.52) towards CRI. However, in the absence of any evidence for the functional status of this polymorphism at present, it would be difficult to substantiate its role in CRI. Val158Met polymorphism has been the most widely implicated for the activity status of COMT. Presence of methionine instead of valine in the protein makes it heat-labile and leads to fourfold reduction in activity [36]. In addition, different studies indicate that this functional polymorphism accounts for most of the variation in peripheral COMT activity [37]. Though, there was no allelic or genotypic association of this SNP (Table 2) in our study, in haplotypic combinations this polymorphism seems to be important. Haplotype 1-1-2-2 [A-C-A (Met)-del C] is predisposing and haplotype 1-1-1-2 [A-C-A (Val)-del C], with only one allele different at Val158Met locus, is protective to CRI. 158Val, the high activity allele of the enzyme, is the predominant allele in our population and is required for regulating normal physiological levels of dopamine. Replacement of Val allele by Met in the haplotype may increase dopamine levels, which is an antagonist to Ang II, and may alter vascular tone and sodium ion balance in kidney. The only other significantly associated haplotype in *COMT *gene, 2-1-2-1 (G-C-A-InsC), is protective towards CRI (Table 3). In the absence of any information on functional status of the other three polymorphisms in this gene, it seems difficult to assess the protective nature of this haplotype.

Out of eight polymorphisms in four dopamine receptor genes (D1–D4), only SNPs *-141 ins/del C and G>A *(1 kb upstream from exon 2) in *DRD2 *gene, showed significant allelic and genotypic association (Table 2), with satisfactory power of the sample size [G = 53% for -141 ins/del C; G = 50.6% for G>A (1 kb upstream from exon 2)]. Odds ratio calculation identified -141 insC allele (OR 2.01, CI 1.35–3.00) and insC/insC genotype (OR 2.19, CI 1.37–3.50) to be predisposing, and insC/delC genotype (OR 0.53, CI 0.33–0.87) of **-**141 ins/del C SNP to be protective to diabetic CRI. Allele 'A' (OR 1.68, CI 1.17–2.42) was found to be predisposing and allele 'G' (OR 0.59, CI 0.41–0.85), and genotype 'GG' (OR 0.52, CI 0.32–0.82) of G>A (1 kb upstream from exon 2) SNP to be protective to CRI. Our result of allelic and genotypic association of *-141 insC/del C *SNP was also reflected in the haplotypic results. The predisposing haplotypes, 1-2-1 (-141 insC-A-T) and 1-2-2 (-141 insC-A-C), contained -141 insC allele and the protective haplotype, 2-2-2 (-141 delC-A-C) contained the -141 delC allele (Table 4). Based on functional analysis it has been reported that -141 delC allele has decreased promoter strength as compared to the -141 ins C allele [38]. Considering the above mentioned facts, it could be argued that -141 ins C allele may lead to excess bio-availability of dopamine, which may alter vascular tone and sodium ion balance in kidney. Function of G>A SNP (I Kb upstream from exon 2) in the gene is not known and thus it's difficult to comment on our observation on this SNP.

There are a few study limitations. Present study is a candidate gene based case-control study and most candidate gene case-control studies of complex traits to date have been disappointing. In diabetic nephropathy alone, many initial reports of significant associations have not withstood the test of replication in other cohorts. Such inconsistencies stem primarily from the very low pre-test probability that any given gene contributes to the susceptibility of a complex trait despite *apriori *hypotheses based on cell, tissue, or animal model experiments. Other reasons include the use of underpowered sample sizes, multiple testing, phenotypic heterogeneity, poor phenotype characterization, selection bias, population stratification, and incomplete knowledge of the complete set of allelic variants in the region of a candidate gene. Our study involves a small sample size. Harrington has commented on standards of evidence in the genomic age and suggests that in a case control study the sample size should be >1000 subjects to arrive at worthwhile conclusions [39]. However, only a very few investigators have access to such a large data and inspite of being a multi-site study the present study could not achieve such large numbers. On the other hand the present study fulfils most of the criteria of a good genetic association study suggested by Hattersley et al [40]. The diagnosis of diabetic renal disease in the present study is based on multiple criteria. Diabetes can produce a large variety of renal lesions and it is likely that molecular and genetic pathways involved in each of these are different. This is the first investigation of association of polymorphisms in the dopaminergic pathway genes with CRI. Significant allelic, genotypic, and haplotypic associations observed with diabetic CRI in this study seem interesting but extrapolation of the results demands replication in a larger sample set.

## Conclusion

It is concluded that polymorphisms *-141 ins/del C *and *G>A *(1 kb upstream from exon 2) in *DRD2*, and *900 ins/del C *in *COMT *genes are associated with susceptibility to diabetic CRI, which if confirmed by replication studies and/or expression analysis would be consistent with a role of dopamine metabolism in disease occurrence. Given that some of the genetic variants were associated with susceptibility and others with protection could be an important indication of the mechanisms of CRI itself. The use of the apposite polymorphisms for prediction or prognosis could well be a subordinate step to that of the confirmation of the observation and of understanding the disease itself.

## Abbreviations

CRI: Chronic renal insufficiency; DM: type-2 diabetes; OR: Odds ratio.

## Competing interests

The author(s) declare that they have no competing interests.

## Authors' contributions

PP was involved in the study design, carried out molecular genetics and statistical analyses, compiled the data, wrote the Ms.; KMPK, ACA, AG, and RG were the principal clinical investigators involved in study design, defining exclusion and inclusion criteria of study subjects and were mainly responsible for identification of study subjects from their respective clinical centres; BKT was the principal geneticist and co-ordinator of the project, involved in conceptualization of the project, study design, oversee complete genetic analyses in the laboratory, critical inputs and finalization of the manuscript.

## Supplementary Material

Additional file 1 Additional file 1. SNPs in *COMT*, *DRD1*, *DRD2*, *DRD3 *and *DRD4 *genes, their location, primer sequences, PCR conditions and restriction enzyme with product sizes. Click here for file

Additional file 2 Additional file 2. SNP haplotypes in DRD4 gene. Click here for file
