# Supplementary material for: Association of dopaminergic pathway gene polymorphisms with chronic renal insufficiency among Asian Indians with type-2 diabetes
Source: BMC Genet. 2008 Mar 22;9:26. doi: 10.1186/1471-2156-9-26 (PMC2287188; doi:10.1186/1471-2156-9-26)
Supplement: Additional file 1 — SNPs in COMT, DRD1, DRD2, DRD3 and DRD4 genes, their location, primer sequences, PCR conditions and restriction enzyme with product sizes. [file 1471-2156-9-26-S1.doc]

Additional file 1

**Supplementary Table 1.** SNPs in *COMT, DRD1, DRD2, DRD3* and *DRD4* genes, their location, primer sequences, PCR conditions and restriction enzyme with product sizes.

| SNPs | Primer sequence | **Annealing temp/**  **restriction enzyme/**  **fragment sizes** |
| --- | --- | --- |
| COMT  -287 G>A | F:5’TAGTAACAGACTGGGCACGAA3’  R:5’GTTCAAAGGGCATTTATCATG3’ | 57ºC /Hind III  G=350  A=276,74 |
| COMT  408 C>G | F:5’CCCTGCACAGGCAAGATCGTG3’  R:5’GCATGCACACCTTGTCCTTCA3’ | 62ºC / Bcl I  C=217  G=105, 112 |
| COMT  472 G > A  (Val158 Met) | F:5’ACTGTGGCTACTCAGCTGTG3’  R:5’CCTTTTTCCAGGTCTGACAA3’ | 57ºC / Nla III  G=25,114,29  A=25,96,18,29 |
| COMT  900Ins/Del C | F:5’GACAACGTGATCTGCCCAGG-3’  R:5’GAGGTGTGCTTTGCATTTAG-3’ | 64ºC / Bgl I  Ins=280  Del=165, 115 |
| DRD1  -48A>G | F:5’GGCTTCTGGTGCCCAAGACAGTG3’  R:5’AGCACAGACCAGCGTGTTCCCCA3’ | 61° C / Dde I  A = 146,42,217  G = 146,259 |
| DRD2  -141 ins/del C | F:5’GACCCAGCCTGCAATCAC3’  R:5’AGGAGCTGTACCTCCTCGG3’ | 57° C / Bst NI  Ins C = 124, 32  Del C = 156 |
| DRD2  G>A  (Intron 1) | F:5’GATGTGTAGGAATTAGCCAGG3’  R:5’GATACCCAGTTCAGGAAGTC3’ | 56° C / Taq 1B  G = 459  A= 267,192 |
| DRD2  T>C  (10 kb downstream from exon 8) | F:5’CCGTCGACGGCTGGCCAAGTTGTCCA 3’  R:5’CCGTCGACCCTTCCTGAGTGTCATCA3’ | 58° C /Taq 1A  T = 310  C =180,130 |
| DRD3  Ser9Gly | F: 5’GCT CTATCT CCA ACT CTC ACA3’  R: 5’ AAG TCT ACT CAC CTC CAG GTA3’ | 55 C/MSc I  C = 304,111,47  G = 206,98,111,47 |
| DRD4  –120bp Deletion | F: 5’GTT GTC TGT CTT TTC TCA TTG TTT CCA TTG 3’  R: 5’GAA GGA GCA GGC ACC GTG AGC 3’ | 61° C  Ins = 549  Del = 429 |
| DRD4  -521 C>T | F: 5’ CGGGGGCTGACCACCAGAGGCTGC 3’  R: 5’ GCATCGACGCCAGCGCCATCCTACC 3’ | 61° C/ Fsp I  C = 285  T = 176,109 |
| DRD4  48 bp VNTR | F: 5’ GCG ACT ACG TGG TCT ACT CG 3’  R: 5’AGG ACC CTC ATG GCC TTG 3’ | 61° C  709,661,613,565,517,459,421,373 |
