# Supplementary material for: Association of dopaminergic pathway gene polymorphisms with chronic renal insufficiency among Asian Indians with type-2 diabetes
Source: BMC Genet. 2008 Mar 22;9:26. doi: 10.1186/1471-2156-9-26 (PMC2287188; doi:10.1186/1471-2156-9-26)
Supplement: Additional file 2 — SNP haplotypes in DRD4 gene. [file 1471-2156-9-26-S2.doc]

## Additional File 2

## Supplementary Table 2. SNP haplotypes in DRD4 gene

| **^a^Haplotype** | **DM** | **CRI** | **χ^2^** | **P** | **O.R (95% CI)** |
| --- | --- | --- | --- | --- | --- |
| 111 | 3 (0.009) | 0 (0.00) | - | - | - |
| 112 | 2 (0.006) | 3 (0.011) | - | - | - |
| 113 | 96 (0.285) | 95 (0.349) | 1.46 | 0.22 | 1.22 (0.88-1.69) |
| 114 | 1 (0.003) | 0 (0.00) | - | - | - |
| 115 | 0 (0.00) | 1 (0.003) | - | - | - |
| 116 | 1 (0.003) | 4 (0.014) | - | - | - |
| 117 | 2 (0.006) | 2 (0.007) | - | - | - |
| 211 | 9 (0.026) | 7 (0.026) | 0.0006 | 0.93 | 0.96 (0.35-2.61) |
| 212 | 1 (0.003) | 0 (0.00) | - | - | - |
| **213** | **22** (0.065) | **8** (0.029) | **4.17** | **0.04** | **0.43 (0.19-0.99)** |
| 121 | 1 (0.003) | 4 (0.015) | - | - | - |
| 122 | 0 (0.00) | 4 (0.015) | - | - | - |
| 123 | 110 (0.327) | 79 (0.29) | 0.96 | 0.32 | 0.84 (0.59-1.19) |
| 124 | 1 (0.003) | 0 (0.00) | - | - | - |
| 125 | 3 (0.009) | 0 (0.00) | - | - | - |
| 126 | 3 (0.009) | 0 (0.00) | - | - | - |
| 127 | 0 (0.00) | 4 (0.015) | - | - | - |
| 221 | 5 (0.015) | 10 (0.036) | 2.99 | 0.08 | 2.53 (0.85-7.48) |
| 222 | 3 (0.009) | 0 (0.00) | - | - | - |
| 223 | 73 (0.217) | 46 (0.169) | 0.09 | 0.75 | 0.93 (0.62-1.40) |
| 272 | 0 (0.00) | 5 (0.018) | - | - | - |

^a^Order of SNPs in the DRD4 haplotypes: –120 bp Deletion- -521 C>T- 48 bp VNTR

Bonferroni’s correction α = 0.0023
